# Influence of Electronic Cigarettes on Antioxidant Capacity and Nucleotide Metabolites in Saliva

**DOI:** 10.3390/toxics9100263

**Published:** 2021-10-14

**Authors:** Dominika Cichońska, Oliwia Król, Ewa M. Słomińska, Barbara Kochańska, Dariusz Świetlik, Jolanta Ochocińska, Aida Kusiak

**Affiliations:** 1 Department of Periodontology and Oral Mucosa Diseases, Medical University of Gdansk, 80-210 Gdansk, Poland; aida.kusiak@gumed.edu.pl; 2 Department of Biochemistry, Medical University of Gdansk, 80-210 Gdansk, Poland; oliwia.krol@gumed.edu.pl (O.K.); ewa.slominska@gumed.edu.pl (E.M.S.); 3 Department of Conservative Dentistry, Medical University of Gdansk, 80-210 Gdansk, Poland; barbara.kochanska@gumed.edu.pl (B.K.); jolanta.ochocinska@gumed.edu.pl (J.O.); 4 Department of Biostatistics and Neural Networks, Medical University of Gdansk, 80-210 Gdansk, Poland; dariusz.swietlik@gumed.edu.pl

**Keywords:** e-cigarettes, antioxidant capacity, saliva, uric acid

## Abstract

The balance between reactive oxygen species production and the activity of antioxidant systems present in saliva is an important element in maintaining oral environment homeostasis. E-cigarettes adversely affect the oral cavity and their cytotoxic effect is related to oxidative stress. The aim of this study was to assess the influence of using electronic cigarettes on antioxidant capacity of saliva. The study involved 110 subjects (35 e-cigarettes users, 33 traditional cigarettes smokers and 42 non-smokers). Laboratory analysis involved quantitation of uric acid, hypoxanthine, xanthine, TAOS (total antioxidant status) and TEAC (Trolox equivalent antioxidant capacity) in saliva. Lower values for TAOS and TEAC were observed among e-cigarettes users and traditional cigarettes smokers in comparison to non-smokers. Uric acid concentration tended to be higher among e-cigarettes users while no differences in hypoxanthine and xanthine saliva concentrations were observed. Electronic cigarettes usage affects antioxidant capacity of saliva to the same extent as traditional cigarettes, when comparing smokers to non-smokers. Further longitudinal studies on a larger study group are needed to assess the effect of changes in antioxidant status on oral health.

## 1. Introduction

Homeostasis in oral environment is mainly provided by saliva, produced constantly by large and small salivary glands [1]. Although 99% of saliva consists of water, it contains also inorganic and organic substances which affect its physicochemical properties [2]. Inorganic components such as sodium, potassium, calcium, magnesium, chlorine, fluorine, iodine, bicarbonates and phosphates are present in saliva in ionic form. Organic saliva components include carbohydrates, lipids, hormones, proteins and non-protein nitrogenous substances. [2]. Individual elements occurring in saliva play a strictly defined role in the proper functioning of the whole organism, nourishing and protecting the surrounding tissues. Saliva glycoproteins moisturize the mucosa and provide protection to oral mucosa against mechanical damage. The presence of buffering bicarbonate and phosphate ions enables to neutralize acids derived from food or that are product of bacterial metabolism, which maintains saliva’s adequate pH value. Saliva contains also salivary amylase, a protein with enzymatic properties and elements presenting antimicrobial activity such as immunoglobulins A, lysozyme, lactoferrin, histamine and leukocytes [2,3]. Moreover, a variety of antioxidants are also present in saliva [4,5,6,7], and the saliva produced by the parotid glands has the highest antioxidant capacity [8].

One of the conditions for maintaining oral environment homeostasis is the balance between ROS (reactive oxygen species) production and the activity of antioxidant systems present in saliva [9]. ROS are mostly generated in cells as a by-product of the mitochondrial electron transport chain, which is dependent on the metabolic status of the cell [10]. Production and effective removal of ROS is crucial in signal transduction, immune defense, matrix remodeling and apoptosis [11]. Low levels of ROS are essential for physiological processes and maintenance of cellular homeostasis [12,13,14]. However, ROS are also an important effector of cell viability control by inducing a cytostatic effect and modulating cell metabolism and gene expression [11]. The excess of free radicals, especially reactive oxygen species, can lead to oxidative stress, which might become the cause of general and local diseases such as periodontitis, diabetes and rheumatoid arthritis [9,15]. Oxidative stress may lead to the destruction of periodontal structures by the degradation of the extracellular matrix of periodontal tissues, and it promotes inflammatory reactions in periodontitis [16,17,18]. Human cells and tissues are protected from the toxic effect of free radicals by special mechanisms including antioxidant enzymatic and non-enzymatic systems. Antioxidant enzymatic systems include peroxidase, catalase, superoxide dismutase and myeloperoxidase, whereas antioxidant non-enzymatic systems include uric acid, reduced glutathione, acute phase proteins, cysteine, ascorbic acid, alpha tocopherol, beta-carotene, retinol and methionine [5,6,7,12,15].

Diverse factors may impact the whole oral environment and composition of saliva is among the most important. Factors that may affect saliva composition include genetic diseases such as Turner syndrome [19], general diseases or tobacco smoking and electronic cigarettes usage [20,21]. Electronic cigarettes are mechanical devices that can be divided into two categories: closed-system and open-system. Closed-system devices tent to resemble traditional cigarettes, are usually disposable and are available in a limited variety of nicotine concentrations and flavors, whereas open-system e-cigarettes are larger in size than traditional cigarettes, can be refilled with e-liquids which are available in a huge variety of flavors and nicotine concentrations and are not disposable after usage [22]. Electronic cigarettes were initially presented as a less harmful substitute for tobacco smoking. However, taking recent research into consideration, this view is controversial [23,24,25,26,27]. It has been proven that electronic cigarettes have a negative effect on oral mucosa leading to death of oral epithelial keratinocytes and periodontal fibroblasts [28,29,30]. The cytotoxic effect is related to oxidative stress and increased concentration of proinflammatory cytokines [30,31]. Chemical compounds found in tobacco smoke and e-cigarettes liquids can dissolve in saliva, leading to disorders in its biochemical composition [21,32,33].

The aim of this study was to assess the influence of electronic cigarettes usage on the antioxidant capacity of saliva. 

## 2. Materials and Methods

### 2.1. Patients’ Population

This study included 110 patients: 35 patients using e-cigarettes (e-cigarettes users), 33 patients smoking traditional cigarettes and 42 non-smoking patients (non-smokers). They were students at the Medical University of Gdansk and young patients, who volunteered for a periodontal examination in the Department of Periodontology and Oral Mucosa Diseases. All participants were generally healthy people aged 20 to 30. Patients with periodontitis and diseases which might interfere condition of oral mucosa like diabetes, disorders of salivary secretion, oral mucosa diseases and people taking medications permanently and treated with antibiotics or steroid preparations in the last 6 months and patients consuming alcoholic beverages were excluded from the research. E-cigarettes users had been using open-system electronic cigarettes with a small nicotine concentration for at least 6 months. Traditional cigarettes smokers were smoking at least 10 cigarettes per day for at least 6 months. People smoking both traditional and electronic cigarettes were not included in this research. The study was conducted in 2018–2019. The study protocol has been approved by the Ethics Committee of Medical University of Gdansk, Poland (NKBBN/161/2014). Ethical aspects of the research followed the World Medical Association Declaration of Helsinki.

### 2.2. Saliva Collection

Mixed unstimulated saliva was collected into a sterile silicon Corning-type test-tube from all patients who participated in this study. Saliva was collected in morning hours, two hours after the last intake of food or drink. Unstimulated salivary samples were obtained by expectoration in absence of chewing movements.

The samples were clarified by centrifugation (2000× *g*; 10 min) and immediately stored for the subsequent determination of uric acid, hypoxanthine, xanthine, TAOS (total antioxidant status) and TEAC (Trolox equivalent antioxidant capacity).

### 2.3. Analysis of Saliva

The whole mixed unstimulated saliva was analyzed in the biochemical laboratory of Conservative Dentistry Medical University of Gdansk and Department of Biochemistry, Medical University of Gdansk, Poland.

To determine nucleotide metabolite concentration, saliva samples were extracted with 1.3 M HClO_4_ (1:1 volume ratio) and centrifuged (20,800× *g*/10 min/4 °C). The supernatants were accumulated and brought to pH 6.0–6.5 using 3 M K_3_PO_4_ solution. After 15-min incubation on ice, samples were centrifuged at (20,800× *g*/10 min/4 °C), and the supernatants were analyzed using high-performance liquid chromatography (HPLC) as we have described previously in detail. 

Determination of sixteen nucleotides, nucleosides and bases was carried out using high-performance liquid chromatography and its application to the study of purine metabolism in hearts for transplantation [34].

Liquid chromatographic evaluation of purine production in the donor human heart during transplantation was performed [35].

The total antioxidant status (TAOS) in saliva was measured by the 2,2′-azino-bis(3-ethylbenzothiazoline-6-sulphonic acid; ABTS) assay, which was based on the capacity of saliva to scavenge the ABTS+ radical. The relative inhibition of ABTS+ formation, after the saliva addition, is proportional to the antioxidant capacity of the sample 1. For the measurement of the total antioxidant status in saliva, 15 µL of saliva was diluted with 180 µL phosphate buffer (0.076 M NaH_2_PO_4_ + 0.23 M Na_2_HPO_4_ in pure water), and then, it was incubated for 10 min at room temperature in a 96-well plate with a 5 µL reaction mixture containing 7 mM ABTS and 2.45 mM potassium persulfate (solved in phosphate buffer: 0.22 M NaH_2_PO_4_ + 0.37 M Na_2_HPO_4_) solved in pure water, pH 7.2. Prior to testing, the reaction mixture was incubated overnight, placing it in the dark at room temperature. The absorbance in the test and control samples (15 μL saline instead of saliva) was read at 630 nm, using a BioTek microplate reader. Results expressed as a percentage inhibition of the reaction were calculated as follows: TAOS [%] = 100 × (Ac−At)/Ac, where Ac is the absorbance of the control sample absorbance and At is the test sample absorbance.

To calculate Trolox equivalent antioxidant capacity (TEAC), a calibration curve for Trolox standard solutions was prepared. Volumes of 15 µL of 30, 100, 500 and 1000 µM Trolox standards were diluted and incubated with reaction mixture in the same manner as in saliva. Antioxidant concentration, as mM Trolox equivalents (TEAC value), in the saliva samples were calculated on the basis of a linear regression equation obtained from the plotted Trolox calibration curve [36].

### 2.4. Statistical Analysis 

The statistical analyses have been performed using the statistical suite StatSoft. Inc. (Tulsa, OK, USA) (2014), STATISTICA (data analysis software system) version 12.0. (2014) from www.statsoft.com and Excel. The significance of the difference between more than two groups was assessed with the one-way analysis of variance (ANOVA Kruskal–Wallis). In the case of statistically significant differences between two groups, post hoc tests were utilized. Correlations were assessed with Pearson and Spearman tests. In all the calculations, the statistical significance level of *p* < 0.001 and *p* < 0.0001 has been used. 

## 3. Results

Table 1 presents the value of uric acid, hypoxanthine, xanthine, uric acid + xanthine, TAOS and TEAC levels on unstimulated saliva among e-cigarette users, cigarette smokers and non-smokers.

**Figure 1:**
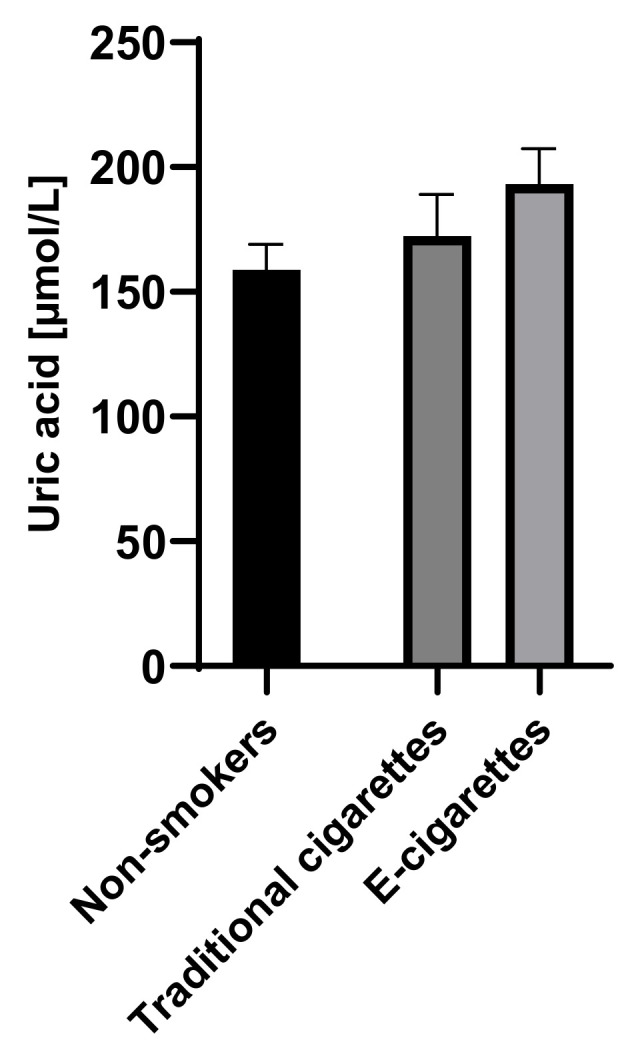
Concentration of uric acid in groups of e-cigarettes users, traditional cigarettes smokers and non-smokers.

The concentration of uric acid among e-cigarettes users was 193.3 µmol/L (14.1); the result in the group of traditional cigarette smokers was 172.4 µmol/L (16.8) and in the group of non-smokers was 158.9 µmol/L (10.3). The concentration of uric acid in group of e-cigarettes users was higher than among traditional cigarettes smokers and non-smokers; however, no statistically significant differences were observed. Saliva concentrations of uric acid are presented in Figure 1.

**Figure 2:**
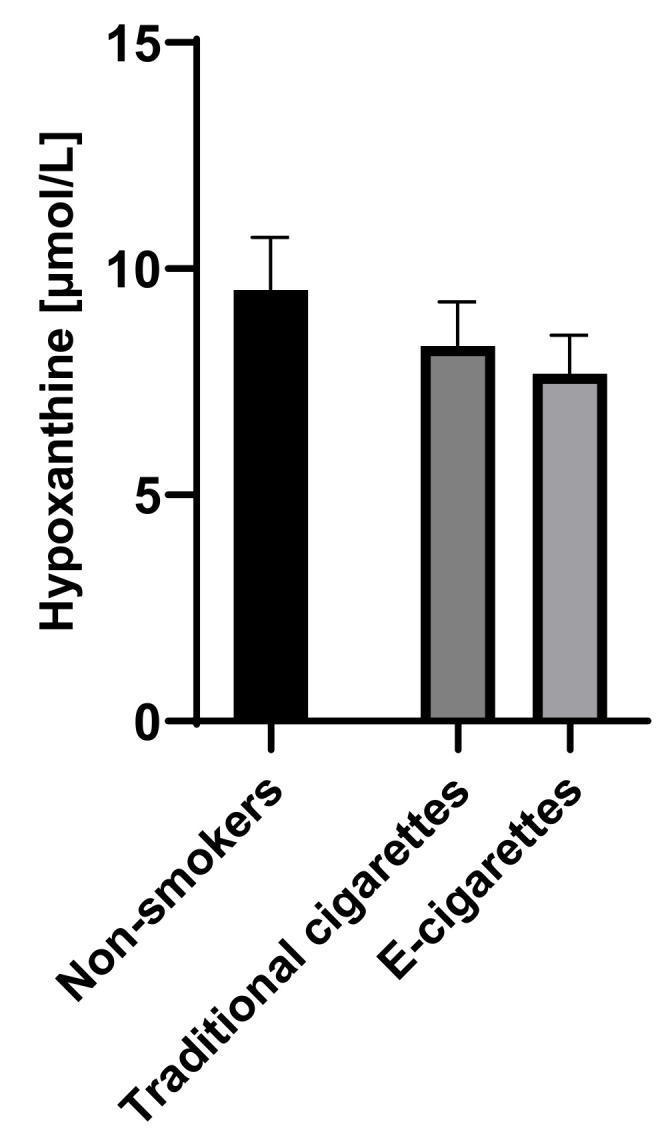
Concentration of hypoxanthine in groups of e-cigarettes users, traditional cigarettes smokers and non-smokers.

The concentration of hypoxanthine in the group of e-cigarettes users was 7.7 µmol/L (0.9), among traditional cigarettes smokers was 8.3 µmol/L (1) and in non-smokers group was 9.5 µmol/L (1.2). Although the concentrations of hypoxanthine among e-cigarettes users were lower than values in the non-smokers group and among traditional cigarettes smokers, no statistically significant differences were observed. Saliva concentrations of hypoxanthine are presented in Figure 2.

**Figure 3:**
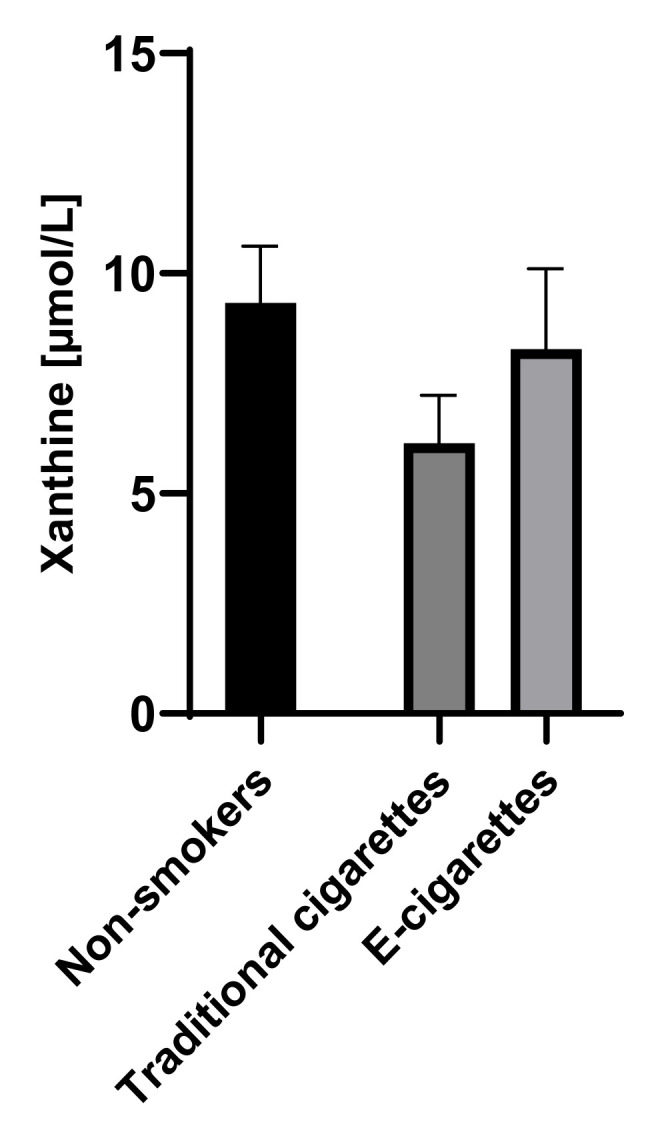
Concentration of xanthine in groups of e-cigarettes users, traditional cigarettes smokers and non-smokers.

The concentration of xanthine in the group of e-cigarettes users was 8.3 µmol/L (1.8), in the group of traditional cigarettes smokers was 6.1 µmol/L (1.1) and among non-smokers was 9.3 µmol/L (1.3). The concentrations of hypoxanthine among e-cigarettes users were higher than the values among traditional cigarettes smokers and lower than the values in the group of non-smokers; therefore, no statistically significant differences were observed. Saliva concentrations of xanthine are presented in Figure 3.

**Figure 4:**
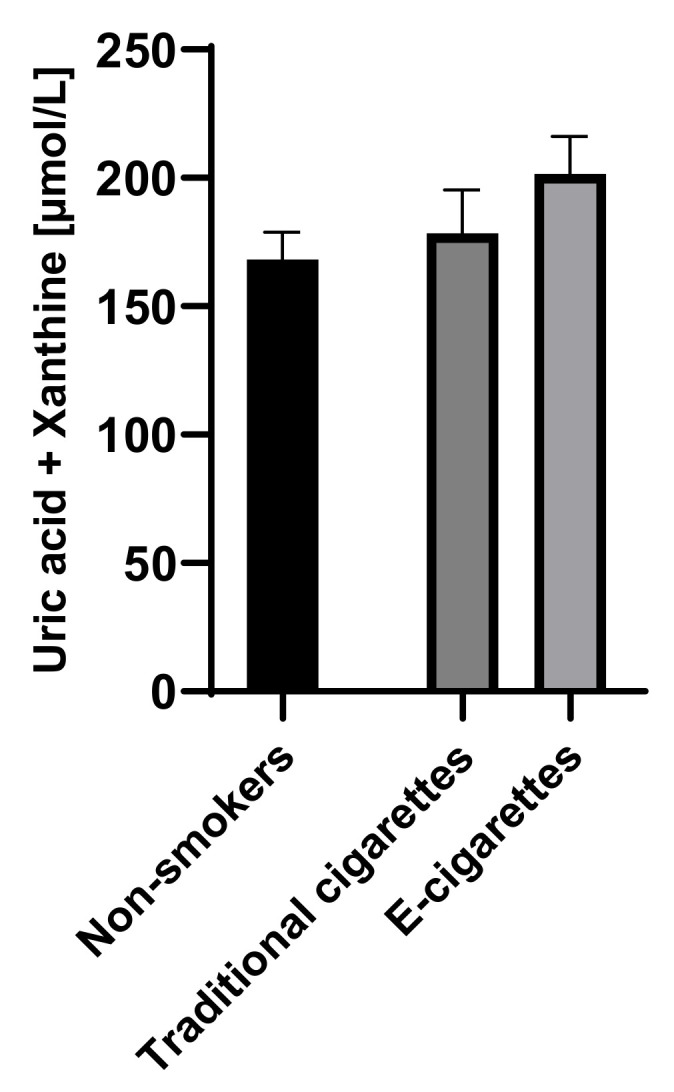
Combined concentrations of uric acid and xanthine in groups of e-cigarettes users, traditional cigarettes smokers and non-smokers.

The combined concentration of uric acid and xanthine among e-cigarettes users was 201.6 µmol/L (14.5), among traditional cigarettes smokers was 178.5 µmol/L (16.8) and among non-smokers was 168.2 µmol/L (10.6). Although the combined concentrations of uric acid and xanthine in the group of e-cigarettes users were higher than among traditional cigarettes smokers and non-smokers, no statistically significant differences were observed. Combined saliva concentrations of uric acid and xanthine are presented in Figure 4.

**Figure 5:**
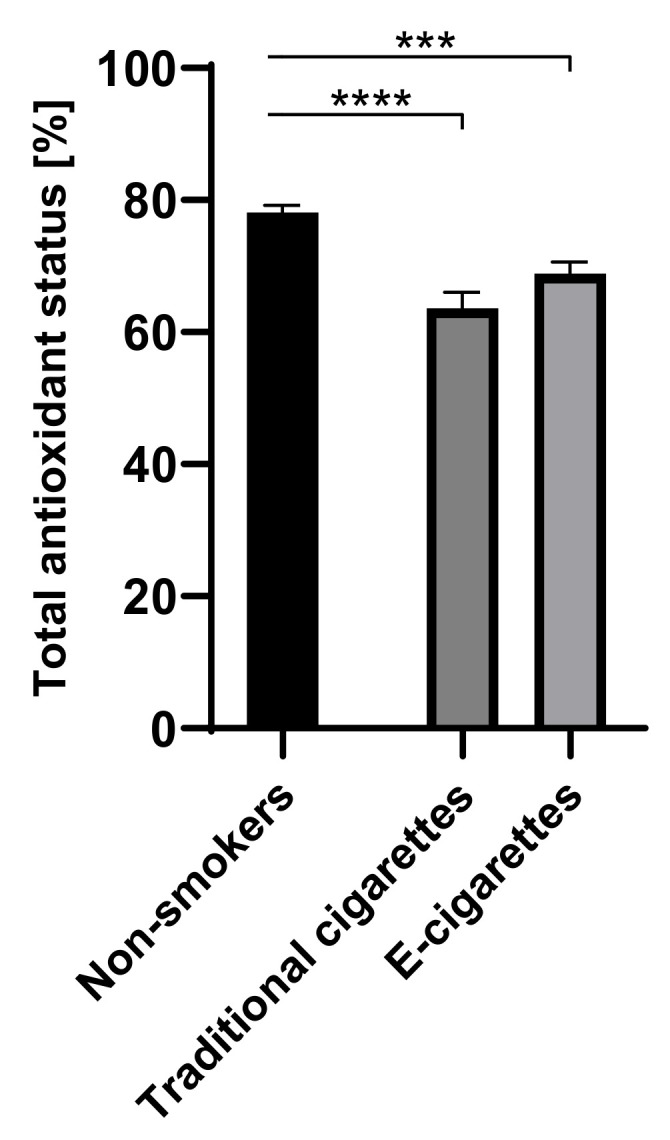
Values of TAOS (total antioxidant status) in groups of e-cigarettes users, traditional cigarettes smokers and non-smokers. *** *p* < 0.001; **** *p* < 0.0001.

The values of TAOS in the group of e-cigarettes users was 68.9% (1.7), among traditional cigarettes smokers was 63.6% (2.4) and among non-smokers was 78.1% (1.1). The values of TAOS in the groups of e-cigarettes users and traditional cigarettes smokers were lower than among non-smokers. Statistically significant differences on the level of *p* < 0.001 were observed among e-cigarettes users in comparison to non-smokers and on the level of *p* < 0.0001 among traditional cigarettes smokers in comparison to non-smokers. Values of TAOS are presented in Figure 5.

**Figure 6:**
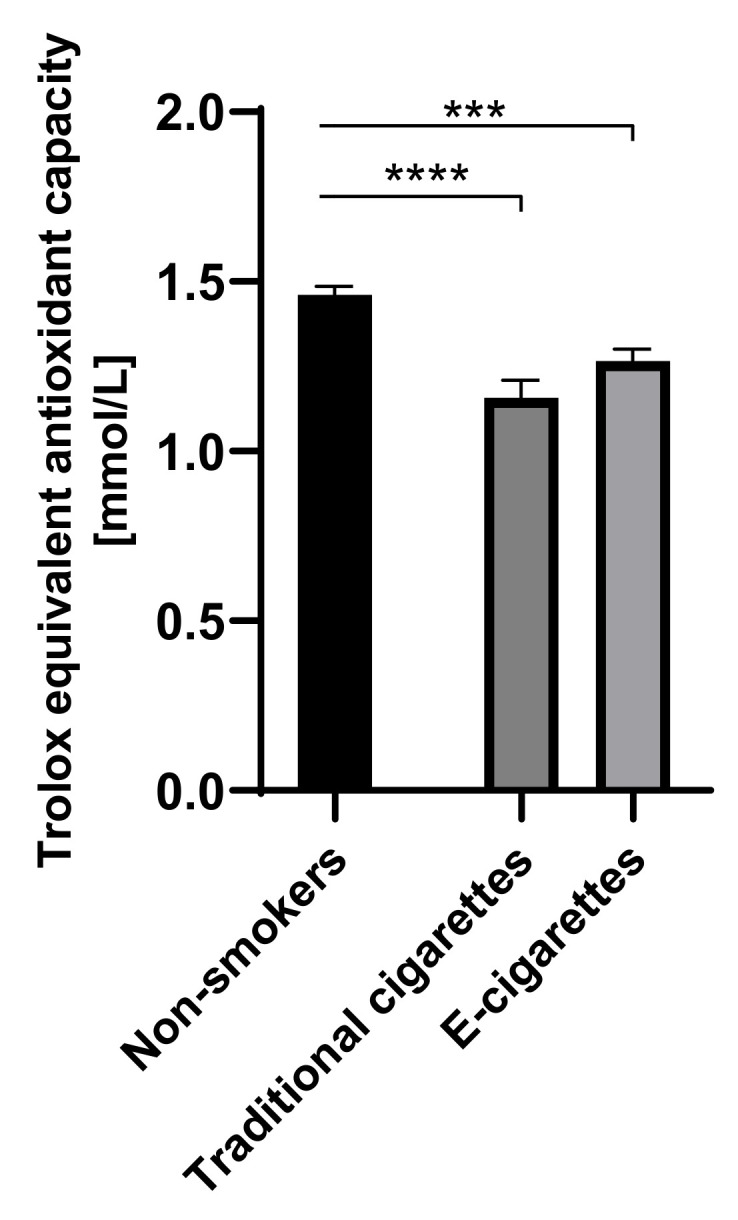
Values of TEAC (Trolox equivalent antioxidant capacity) in groups of e-cigarettes users, traditional cigarettes smokers and non-smokers. *** *p* < 0.001; **** *p* < 0.0001.

The value of TEAC in the group of e-cigarettes users was 1.3 mM (0.04), in the group of traditional cigarettes users was 1.2 mM (0.05) and among non-smokers was 1.5 mM (0.03). The values of TEAC among e-cigarettes users and traditional cigarettes smokers were lower than in the non-smokers group. Statistically significant differences on the level of *p* < 0.001 were observed in the group of e-cigarettes users compared to the non-smokers and on the level of *p* < 0001 between traditional cigarettes smokers and non-smokers. Values of TEAC are presented in Figure 6.

**Figure 7:**
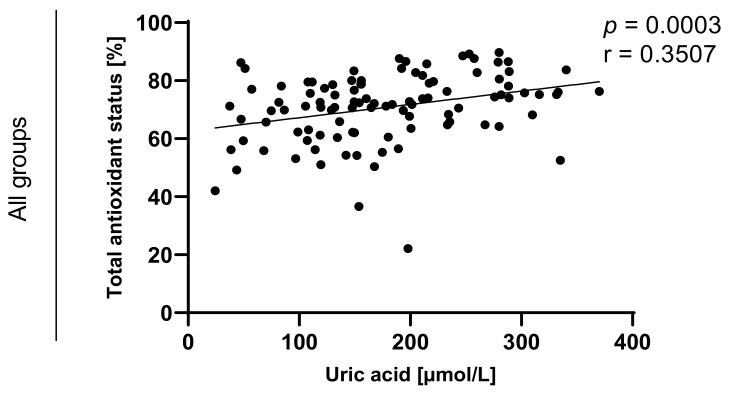
Correlation between uric acid and TAOS (total antioxidant status) among e-cigarettes users, traditional cigarettes smokers and non-smokers.

**Figure 8:**
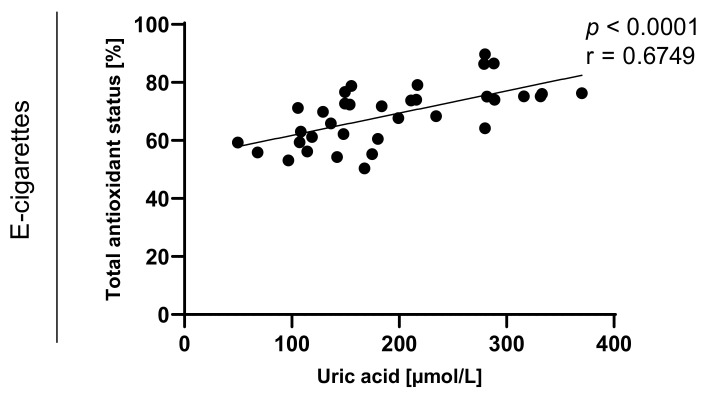
Correlation between uric acid and TAOS (total antioxidant status) among e-cigarettes users.

**Figure 9:**
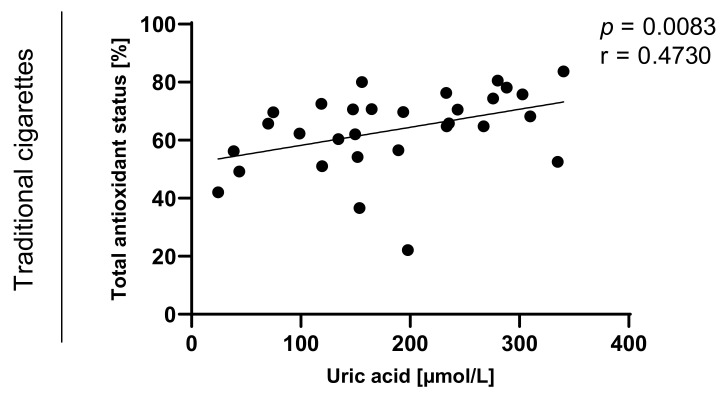
Correlation between uric acid and TAOS (total antioxidant status) among traditional cigarettes smokers.

**Figure 10:**
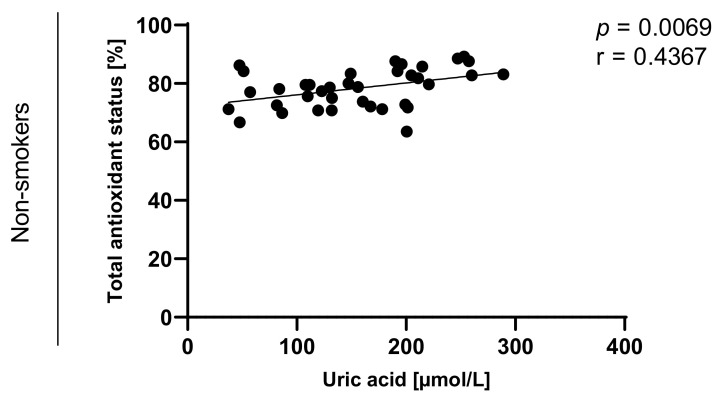
Correlation between uric acid and TAOS (total antioxidant status) among non-smokers.

Statistically significant correlations between values of TAOS on level of *p* < 0.0003 in the group of e-cigarette users and on the level of *p* < 0.0001 in the group of traditional cigarettes smokers were also observed and are presented in Figure 7, Figure 8, Figure 9 and Figure 10.

## 4. Discussion

The most important finding of this study was determining that the oxidant status of saliva is reduced by use of electronic cigarettes to the same extent as it is by traditional cigarettes smoking. This highlights the important risk of the adverse effects of electronic cigarettes, in contrast to the common view of their limited toxicity.

Saliva is the first body fluid that has a direct contact with both tobacco smoke and electronic cigarettes vapor and is in the first line in antioxidant defense [5]. Tobacco smoke is a complex mixture of chemical compounds, which are a source of free radicals and oxidants causing adverse side effects in the oral cavity [37,38]. Tobacco smoking might be the reason for an adaptive response, consequently leading to an increase in the antioxidant levels in saliva or to a decrease in saliva antioxidant defenses [39]. Liquids used in electronic cigarettes mainly consist of propylene glycol, glycerin, nicotine and flavor additives [23,24,25]. However, e-liquids heated to high temperatures might become a source of detectible levels of potentially harmful chemicals as formaldehyde, acrolein, heavy metals and acetaldehyde carbonyls [40,41]. Oxidants or reactive oxygen species are also generated by vaporizing e-liquids, which are influenced by the heating element of the electronic cigarette and associated with e-liquid flavor. The aerosol generated by electronic cigarettes might pose an impact on levels of oxidative stress [42]; however, it has not been proven yet which electronic cigarettes’ factors might be related to oxidative stress generation [43].

Uric acid (UA) is the most important non-enzymatic antioxidant present in saliva. This is a plasma born antioxidant that facilitates removal of hydroxyl radical and superoxide anion [6,44]. Increased concentration of uric acid in saliva might reflect a response to oxidative stress and be related to periodontitis or cancer [44,45,46]. Our results indicate that uric acid concentration in the saliva of e-cigarettes users tended to be higher than among traditional cigarettes smokers and non-smokers. Studies conducted by Kodakova et al. [39] and Zappacosta et al. [4] indicated no differences in the level of uric acid in saliva between traditional cigarettes smokers and non-smokers. On the contrary, Greabu et al. [47], Ahmadi-Motamayel [48] and Abdolsamadi et al. [49] observed the decreased uric acid levels in the saliva of traditional cigarettes smokers compared to non-smokers. Our results show little effect of traditional cigarette smoking on uric acid concentration in saliva. The trend towards an increase in uric acid concentration in e-cigarettes users may indicate facilitated transport of uric acid from blood into saliva, and this effect is worth further investigation. 

Hypoxanthine and xanthine are products of purine metabolism. Adenine nucleotides could be converted in several steps into hypoxanthine, which is then transformed to xanthine and then to uric acid [50]. Our results indicated that saliva concentration of hypoxanthine and xanthine in electronic cigarettes users and traditional cigarettes smokers tended to be lower than among non-smokers. Concentrations of hypoxanthine among traditional cigarettes were higher and that of xanthine lower in comparison to e-cigarettes users. Such pattern together with uric acid concentration changes highlights a shift towards uric acid concentration either by accelerated breakdown of hypoxanthine or xanthine or due to increased transport of uric acid. Our study is the first analysis of the impact of e-cigarettes usage and of smoking traditional cigarettes on the levels of hypoxanthine and xanthine in saliva. 

Total antioxidant status (TAOS) is the sum of all antioxidants present in saliva, and uric acid makes up to 85% of the TAOS [5]. Measurement of TAOS value reflects the current efficiency of antioxidant mechanisms. Initially, during exposure to oxygen free radicals, an adaptive increase in TAOS value was observed, while sustained exposure to oxygen free radicals led to a decrease in the concentration of antioxidants, which resulted in a decrease in the TAOS value [51]. In our research TAOS in the saliva of e-cigarettes users and traditional cigarettes smokers was lower than among non-smokers. Hamo Mahmood et al. also observed the decrease of TAOS among traditional cigarettes smokers in comparison to non-smokers [52]. Research conducted by Bakhtiari et al., on the TAOS in the saliva of traditional cigarettes smokers also demonstrated lower values than among non-smokers [53]. Kodakova et al. [39] and and Zappacosta et al. [4] reported no differences in levels of TAOS in saliva between traditional cigarettes smokers and non-smokers. However, Greabu et al. [47], Ahmadi-Motamayel [48] and Nagler [54] observed the increase of TAOS in the saliva of traditional cigarettes smokers compared to non-smokers. According to Hamo Mahmood et al., the reduction of TAOS among traditional cigarettes smokers might be related to the exhaustion of saliva antioxidants caused by the presence of high amounts of free radicals in cigarette smoke, which may lead to an oxidative stress [52]. 

Trolox equivalent antioxidant capacity (TEAC) enables to measure total antioxidant capacity of saliva by assessing the capacity of a compound to scavenge ABTS radicals [55,56]. In our research, the values of TEAC among e-cigarettes users and traditional cigarettes smokers were lower than in non-smokers group. Statistically significant differences were observed between both e-cigarettes users compared to non-smokers and traditional cigarettes smokers compared to non-smokers. Research on the impact of e-cigarettes and traditional cigarettes on TEAC has not been published yet. 

The salivary antioxidant system is relevant when considering saliva’s anti-cancer capacity and protection from development of periodontal diseases [9,46,56]. Among patients with periodontitis, a decreased efficiency of antioxidant mechanisms has been observed [9]. Konopka et al., demonstrated lower values of TAOS in saliva among patients with periodontitis as compared to control group [9]. The decreased values of TAOS in saliva might be related to the depletion of antioxidants as a result of a chronic inflammation. The connection between periodontitis and the antioxidant potential of saliva is also confirmed by a positive correlation between the concentration of uric acid in saliva and the parameters of periodontal tissues inflammation [9]. Disorders of antioxidant potential are also strictly related to the risk of oral cancer development. Free radicals and ROS can induce DNA damage, which may lead to cancerous transformation. Those negative effects of ROS are counteracted by antioxidants [57].

## 5. Conclusions

Electronic cigarettes usage adversely affects the antioxidant capacity of saliva, in comparison to non-smokers, to the same extent as smoking traditional cigarettes. This might present an important clinical risk of oral cavity disorders. Further longitudinal studies on a larger group should be conducted in order to assess how the changes observed in the antioxidant capacity of saliva translate to oral health.

## Figures and Tables

**Table 1:** Mean values of uric acid, hypoxanthine, xanthine, uric acid + xanthine, TAOS and TEAC on unstimulated saliva among e-cigarette users, cigarette smokers and non-smokers.

Groups	UA [µmol/L]	Hx [µmol/L]	X [µmol/L]	UA + X [µmol/L]	TAOS [%]	TEAC [mM]
	$\bar{X}$ (SEM)	$\bar{X}$ (SEM)	$\bar{X}$ (SEM)	$\bar{X}$ (SEM)	$\bar{X}$ (SEM)	$\bar{X}$ (SEM)
E-cigarettes users	193.3 (14.1) *n* = 35	7.7 (0.9) *n* = 35	8.3 (1.8) *n* = 35	201.6 (14.5) *n* = 35	68.9 (1.7) ^a^ *n* = 35	1.3 (0.04) ^c^ *n* = 35
Cigarettes smokers	172.4 (16.8) *n* = 33	8.3 (1) *n* = 33	6.1 (1.1) *n* = 33	178.5 (16.8) *n* = 33	63.6 (2.4) ^b^ *n* = 31	1.2 (0.05) ^d^ *n* = 31
Non-smokers	158.9 (10.3) *n* = 42	9.5 (1.2) *n* = 42	9.3 (1.3) *n* = 42	168.2 (10.6) *n* = 42	78.1 (1.1) ^a,b^ *n* = 38	1.5 (0.03) ^c,d^ *n* = 38

Legend: UA—uric acid, Hx—hypoxanthine, X—xanthine, TAOS—total antioxidant status, TEAC—Trolox equivalent antioxidant capacity, mean values, SEM—standard error of mean; a,b,c,d—testify to statistically significant values; ^a-a, b-b, c-c, d-d^—groups with statistical significance, *p* < 0.001 for ^a-a, b-b^, *p* < 0.0001 for ^c-c, d-d^.
